# Association Between Coffee and Caffeine Intake and Risk of Breast Cancer: A Systematic Review and Meta‐Analysis of Cohort Studies

**DOI:** 10.1002/hsr2.72580

**Published:** 2026-06-03

**Authors:** Mehdi Karimi, Shadi Ghaemi, Razieh Goudarzi, Mohammad Amin Karimi, Kimia Kazemi, Hoda Haghshenas, Omid Asbaghi

**Affiliations:** ^1^ Faculty of Medicine Bogomolets National Medical University Kyiv Ukraine; ^2^ Department of Community Nutrition School of Nutritional Sciences and Dietetics, Tehran University of Medical Sciences (TUMS) Tehran Iran; ^3^ Clinical Nutrition and Dietetics Department, Faculty of Nutrition Sciences and Food Technology National Nutrition and Food Technology Research Institute, Shahid Beheshti University of Medical Sciences Tehran Iran; ^4^ School of Medicine Shahid Beheshti University of Medical Sciences Tehran Iran; ^5^ Department of Food Science and Technology Islamic Azad University ‐ Ayatollah Amoli Branch Amol Iran; ^6^ Faculty of Medicine Jahrom University of Medical Sciences (JUMS) Jahrom Iran; ^7^ Cancer Research Center Shahid Beheshti University of Medical Sciences Tehran Iran

**Keywords:** breast cancer, caffeine, coffee, decaffeinated, incidence, meta‐analysis, risk

## Abstract

**Background and Aims:**

Coffee and caffeine consumption have been widely investigated for their potential effects on cancer risk, driven by their bioactive compounds and antioxidant properties. However, findings on the association between them and breast cancer risk remain inconsistent across studies. This study aimed to conduct a systematic review and meta‐analysis to examine the association between coffee, decaffeinated coffee, and caffeine intake and the risk of breast cancer.

**Methods:**

A literature search was conducted in databases from inception through 1st February 2026 to identify relevant cohort studies. Meta‐analyzes were performed using the RRs with 95% confidence intervals (95% CI) as effect measures.

**Results:**

Thirty‐one cohort studies were included. Regular coffee consumption was associated with a modest reduction in breast cancer risk among women (pooled HR: 0.95; 95% CI: 0.91–0.99; *p* = 0.035), particularly in postmenopausal populations. In contrast, no significant associations were observed for caffeine intake (HR: 0.99; 95% CI: 0.94–1.04; *p* = 0.824) or decaffeinated coffee consumption (HR: 1.00; 95% CI: 0.94–1.07; *p* = 0.793). Subgroup analyzes by menopausal status, cohort duration, and hormone receptor subtype yielded consistent null results across both caffeine‐ and decaffeinated‐coffee categories.

**Conclusion:**

This meta‐analysis indicates that while caffeine and decaffeinated coffee intake are not significantly associated with breast cancer risk, regular coffee consumption may offer a modest inverse association, especially among postmenopausal women. The findings highlight the potential influence of bioactive compounds in coffee beyond caffeine on breast cancer development. Further large‐scale prospective studies are needed to substantiate these results and elucidate underlying biological pathways.

## 1 Introduction

Breast cancer remains the most frequently diagnosed malignancy and a leading cause of cancer‐related mortality among women worldwide [1]. Data from the Global Burden of Disease (GBD) study indicate that the incidence of breast cancer among women aged 15–49 increased by approximately 120% between 1990 and 2021, with a further 47% rise projected by 2040. According to Cancer Research UK and the World Health Organization (WHO), breast cancer accounts for nearly 15% of all newly diagnosed cancers in women, representing more than 2.3 million cases annually worldwide [2, 3]. Although survival rates have improved substantially in high‐income countries, significant disparities in incidence, mortality, and access to care persist across geographic and socioeconomic settings [4, 5, 6].

Breast cancer is a multifactorial disease influenced by genetic [7], hormonal, environmental, and lifestyle factors. Fewer than 10% of cases are due to inherited mutations, while most are linked to modifiable risks such as obesity, alcohol use, reproductive factors, and exogenous hormones [3]. Emerging evidence also indicates that antipsychotic medications may elevate breast cancer risk in women, possibly through prolactin‐induced hormonal dysregulation [8]. Within this intricate etiological landscape, increasing research has focused on dietary and lifestyle determinants as potentially modifiable contributors to breast cancer susceptibility. In particular, dietary patterns such as adherence to the Mediterranean diet [9], as well as high consumption of fast foods and ultra‐processed products [10], have garnered attention. Beverage‐related exposures, especially coffee and caffeine intake, have also been extensively investigated for their potential role in breast cancer risk [11, 12].

Coffee is among the most widely consumed beverages worldwide, with global consumption exceeding two billion cups per day [13, 14]. Beyond its well‐known stimulant, caffeine, coffee contains more than 1000 biologically active constituents, including chlorogenic acids, lignans, polyphenols, diterpenes such as cafestol and kahweol, and melanoidins, which exert antioxidant, anti‐inflammatory, and potential anticarcinogenic effects [15]. Coffee‐derived bioactive compounds modulate critical molecular pathways that regulate xenobiotic metabolism, oxidative stress responses, and cellular defense mechanisms. Experimental evidence indicates that these constituents can restore redox balance, induce apoptosis, and inhibit DNA damage and uncontrolled proliferation across various cancer models. Notably, emerging data suggest sex‐specific variability in these effects, with stronger protective associations among women, possibly due to hormonal factors and differences in caffeine metabolism [14]. Both experimental and epidemiological studies suggest that coffee may exert chemopreventive effects across several malignancies, particularly liver and colorectal cancers [16]. Recent epidemiological evidence further indicates that coffee consumption is not significantly associated with an increased risk of thyroid cancer [17] or erectile dysfunction [18], supporting its overall safety profile in relation to these outcomes. The potential protective role of coffee has been attributed to its antioxidant and anti‐inflammatory properties, as well as its ability to enhance insulin sensitivity and modulate estrogen metabolism—mechanisms that provide biologically plausible pathways for reducing breast cancer risk. Mechanistically, bioactive compounds in coffee, including caffeine and polyphenols, may influence cytochrome P450 enzyme activity and estrogen hydroxylation, processes implicated in the pathogenesis of hormone receptor–positive tumors [19].

Epidemiological evidence on coffee and breast cancer risk remains mixed. While some large cohorts and meta‐analyzes have reported inverse associations between high coffee intake and postmenopausal breast cancer [20, 21], others have found no significant relationship after adjustment for key confounders [11, 22]. Epidemiologic evidence comparing caffeinated and decaffeinated coffee remains limited and inconsistent: some studies report no association for either type [23], whereas others suggest a modest inverse relationship for caffeinated, but not decaffeinated coffee [20, 24].

Despite extensive investigation, important gaps remain in the current literature. Existing studies have reported inconsistent findings, and many prior meta‐analyzes have not comprehensively differentiated between total, caffeinated, and decaffeinated coffee intake, nor have they adequately explored potential effect modification by menopausal status and geographic region. Moreover, recent large‐scale cohort studies have not been fully integrated into earlier syntheses, thereby limiting the timeliness and precision of the current evidence. Therefore, the present systematic review and meta‐analysis was conducted to address these gaps by providing an updated and comprehensive evaluation of the associations between total coffee, caffeinated and decaffeinated coffee, and caffeine consumption with breast cancer risk, along with stratified analyzes to clarify potential subtype‐specific and population‐specific effects.

## 2 Method

### 2.1 Study Protocol, Design, and Framework

This study was conducted in accordance with the PRISMA (Preferred Reporting Items for Systematic Reviews and Meta‐Analyzes) statement [25] and structured using the PICOS framework [26], as follows:

‐ P (Population): Adult women
‐ I (Intervention/Exposure): coffee (either caffeinated or decaffeinated), and caffeine intake
‐ C (Comparison): highest versus lowest coffee and caffeine intake
‐ O (Outcomes): risk of breast cancer
‐ S (Study Design): Cohort studies

### 2.2 Search Strategy

A comprehensive, systematic literature search was conducted to identify relevant cohort studies examining the association between coffee or caffeine consumption and breast cancer risk. Three major electronic databases (PubMed, Scopus, and Web of Science) were searched for studies published up to February 1st, 2026. The search strategy combined Medical Subject Headings (MeSH) and free‐text terms related to both the exposure and outcome of interest, using Boolean operators (AND/OR) to ensure optimal sensitivity and completeness. The exact query words and search strategy, including: (“coffee” OR “caffeine” OR “caffeinated” OR “decaffeinated”) AND (“Breast cancer” OR “Breast malignancy” OR “Breast carcinoma” OR “Breast adenocarcinoma” OR “Breast tumor”), applied to titles and abstracts. To enhance coverage, manual searches were also conducted through Google Scholar, the reference lists of all included articles, and relevant systematic reviews to identify additional eligible studies not captured by the base search. The complete search syntax and database‐specific strategies are provided in Suppoerng Information Tables S1 and S2.

### 2.3 Eligibility Criteria

The inclusion criteria for this meta‐analysis were as follows [1]: studies examining the association between caffeine and/or coffee consumption (including caffeinated or decaffeinated coffee) and the risk of breast cancer in adult women [2]; original cohort studies with a prospective or retrospective design [3]; studies officially published in peer‐reviewed journals [4]; studies published in the English language; and [5] studies reporting sufficient quantitative data to calculate or extract effect size estimates, such as hazard ratios (HR), relative risks (RR), or odds ratios (OR), along with their corresponding 95% confidence intervals (CI).

The exclusion criteria were defined as [1]: interventional or experimental studies [2]; review articles of any type, including systematic reviews, meta‐analyzes, and narrative reviews [3]; conference abstracts or unpublished reports [4]; animal or laboratory‐based studies [5]; studies lacking sufficient data for effect size calculation or extraction [6]; studies not specifically focused on breast cancer or its subtypes as the primary outcome [7]; studies in which coffee or caffeine consumption was not the primary exposure variable; and [8] studies presenting significant methodological flaws that could compromise validity.

### 2.4 Study Selection Process

Two independent reviewers (SH, GH, and R.G.) conducted a systematic screening of all identified records using a two‐stage approach. Following the database searches, all retrieved records were imported into *EndNote* software to manage references and remove duplicates. Initially, titles and abstracts were evaluated for potential eligibility. Studies meeting these preliminary criteria proceeded to full‐text assessment to confirm inclusion. Any disagreements at either stage were resolved through discussion, and persistent discrepancies were referred to the project supervisor (M.K.) for a final decision. This rigorous process ensured that only studies relevant to the association between coffee consumption and breast cancer risk were included in the meta‐analysis.

### 2.5 Data Extraction

After study selection, two reviewers (SH, GH, and R.G.) independently extracted data from the included studies. Extracted information encompassed study characteristics (first author, publication year, country, study design, sample size, participant age, and follow‐up duration), exposure details (caffeinated or decaffeinated coffee), outcome measures (breast cancer diagnosis), and effect estimates (HR, OR, or RR with 95% CI, including adjustment variables). For each study, the effect size comparing the highest versus the lowest coffee consumption, using the most fully adjusted and non‐overlapping estimate, was recorded. Any discrepancies during data extraction were resolved through discussion or, if necessary, consultation with the third reviewer (M.K.). The finalized dataset was organized in an Excel spreadsheet and prepared for statistical analysis to ensure consistency and comparability across studies. Subgroup analyzes by menopausal status, hormone receptor subtype, cohort duration, and geographic region were conducted in accordance with the literature.

### 2.6 Quality Assessment

The methodological quality of the included cohort studies was assessed using the Newcastle–Ottawa Scale (NOS). This tool evaluates observational studies across three domains: selection of cohorts (maximum 4 stars), comparability (maximum 2 stars), and outcome assessment (maximum 3 stars), with a total possible score of nine stars. One star for comparability was awarded for adjustment for age, and an additional star for adjustment for major breast cancer risk factors, including body mass index, menopausal status, reproductive factors, hormone therapy use, smoking, alcohol intake, or physical activity. Outcome assessment was considered adequate if breast cancer cases were ascertained through cancer registries or medical records, follow‐up duration was ≥ 5 years, and loss to follow‐up was < 20% or was accounted for through registry linkage. Two reviewers independently conducted the quality assessment and resolved disagreements by consensus. Studies were classified as high quality (7–9 stars), moderate quality (4–6 stars), or low quality (0–3 stars).

### 2.7 Statistical Analysis

All statistical analyzes were performed using **Stata version 11.0** (StataCorp, College Station, TX, USA). We pooled the most fully adjusted effect estimates from each study, including HRs, RRs, and ORs, comparing the highest versus lowest categories of coffee consumption (regular and decaffeinated) and caffeine intake in relation to breast cancer risk. Given the relatively low incidence of breast cancer, ORs and HRs were considered reasonable approximations of RRs under the rare disease assumption. Accordingly, all estimates were treated as measures of relative risk and combined on the logarithmic scale.

Summary effect estimates and corresponding 95% confidence intervals (CIs) were calculated using a random‐effects model based on the DerSimonian and Laird method, incorporating both within‐ and between‐study variability [27]. Study‐specific weights were assigned using the inverse‐variance method based on log‐transformed effect estimates and their standard errors. Statistical heterogeneity was evaluated using Cochran's Q test and quantified with the I^2^ statistic, with values of approximately 25%, 50%, and 75% representing low, moderate, and high heterogeneity, respectively [28].

When studies reported multiple eligible estimates, those derived from mutually exclusive subgroups (e.g., menopausal status, hormone receptor subtype, or type of coffee) were included as independent comparisons in subgroup analyzes. However, when multiple models were reported for the same population, only the most fully adjusted estimate was selected to avoid duplication and statistical dependence.

Prespecified subgroup analyzes were conducted according to menopausal status, cohort duration, geographic region, and hormone receptor subtype (ER/PR status) to explore potential sources of heterogeneity. Sensitivity analyzes were performed using a leave‐one‐out approach to assess the robustness of the pooled estimates. Publication bias was evaluated by visual inspection of funnel plots and formally tested using Begg's rank correlation and Egger's regression asymmetry tests. All statistical tests were two‐sided, and *p*‐values < 0.05 were considered statistically significant. Effect estimates are presented with 95% CIs.

## 3 Results

### 3.1 Study Selection

**Figure 1:**
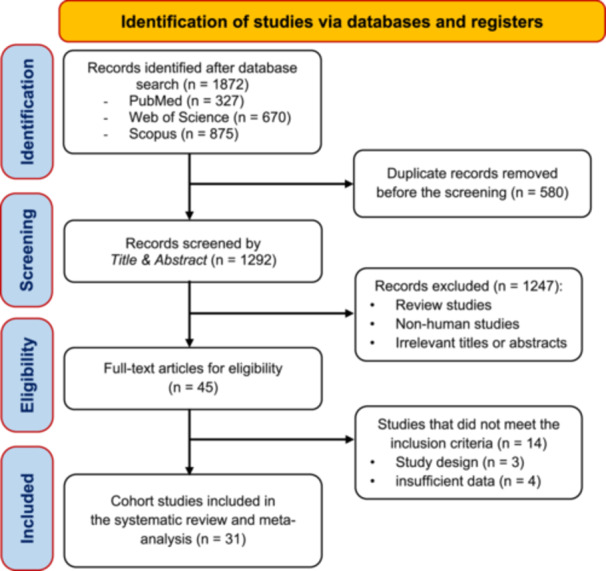
PRISMA flow chart of the study selection process in the systematic review.

A total of 1872 records were initially identified through searches of three electronic databases: PubMed (*n* = 327), Web of Science (*n* = 670), and Scopus (*n* = 875). After removing 580 duplicate entries, 1292 unique records remained for title and abstract screening. Of these, 1247 were excluded for failing to meet the eligibility criteria or for being unrelated to the research objective. The full texts of the remaining 45 articles were then assessed for eligibility, resulting in the exclusion of 14 studies due to insufficient data, irrelevance, or lack of specific effect estimates for breast cancer risk. Ultimately, 31 cohort studies [20, 29, 30, 31, 32, 33, 34, 35, 36, 37, 38, 39, 40, 41, 42, 43, 44, 45, 46, 47, 48, 49, 50, 51, 52, 53, 54, 55, 56, 57, 58] satisfied all inclusion criteria and were included in the final systematic review and meta‐analysis (Figure 1).

### 3.2 Characteristics of Included Studies

Characteristics of the included 31 cohort studies in the systematic review and meta‐analysis examining the association between caffeine, caffeinated coffee, and decaffeinated coffee intake and breast cancer risk are summarized in Table 1.

**Table 1:** Characteristics of the included 31 cohort studies in the systematic review and meta‐analysis examining the association between caffeine, caffeinated coffee, and decaffeinated coffee intake and breast cancer risk.

Study	Country	Study design	Duration	Total sample size	Exposure sample size	Age (years)	Exposure	Types of arms	Types of ES	Effect size (95%CI)
**Caffeine**										
Folsom A. R. et al. [1993] [1]	USA	Cohort	1986–1990	34,388	580	62 ± 4 [55–69]	Caffeine	Dose	RR	1.02 (0.78, 1.33)
Michels et al. [2002] [2]	Sweden	Cohort, Prospective	1987–1990	59,036	1271	52	Caffeine	Dose & 25 ≥ BMI	HR	1.06 (0.83, 1.34)
Michels et al. [2002] [2]	Sweden	Cohort, Prospective	1987–1990	59,036	1271	52	Caffeine	Dose & 25 < BMI	HR	1.04 (0.79, 1.37)
Ganmaa. D. et al. [2008] [3]	USA	Cohort, Prospective	1980–2002	85,987	5272	30–55	Caffeinated	Dose & ER−/PR−	RR	0.88 (0.69, 1.12)
Ganmaa. D. et al. [2008] [3]	USA	Cohort, Prospective	1980–2002	85,987	5272	30–55	Caffeinated	Dose & ER + /PR+	RR	0.88 (0.77, 1.00)
Ishitani. K. et al. [2008] [4]	USA	Cohort, Prospective	1992–2004	38,432	1188	≥ 45	Caffeine	Dose & ER − PR−	RR	1.68 (1.01, 2.81)
Ishitani. K. et al. [2008] [4]	USA	Cohort, Prospective	1992–2004	38,432	1188	≥ 45	Caffeine	Dose & ER + PR−	RR	1.38 (0.78, 2.46)
Ishitani. K. et al. [2008] [4]	USA	Cohort, Prospective	1992–2004	38,432	1188	≥ 45	Caffeine	Dose & ER + PR+	RR	0.84 (0.67, 1.06)
Boggs D. A. et al. [2010] [5]	USA	Cohort, Prospective	1995–2007	52,062	1268	21–69	Caffeine	Dose & Postmenopausal	IRR	1.03 (0.79, 1.34)
Boggs D. A. et al. [2010] [5]	USA	Cohort, Prospective	1995–2007	52,062	1268	21–69	Caffeine	Dose & Premenopausal	IRR	0.97 (0.74, 1.26)
Fagherazzi. G. et al. [2011] [6]	France	Cohort, Prospective	1993–2005	67,703	2868	40–65	Caffeine	Dose & ER − PR−	HR	1.01 (0.77, 1.16)
Fagherazzi. G. et al. [2011] [6]	France	Cohort, Prospective	1993‐2005	67,703	2868	40–65	Caffeine	Dose & ER − PR+	HR	0.66 (0.39, 1.16)
Fagherazzi. G. et al. [2011] [6]	France	Cohort, Prospective	1993–2005	67,703	2868	40–65	Caffeine	Dose & ER + PR−	HR	1.20 (0.94, 1.26)
Fagherazzi. G. et al. [2011] [6]	France	Cohort, Prospective	1993–2005	67,703	2868	40–65	Caffeine	Dose & ER + PR+	HR	0.99 (0.86, 1.16)
Oh et al. [2015] [7]	Sweden	Cohort, Prospective	1992–2035	42,099	1395	30–76	Caffeinated	Postmenopausal	RR	0.86 (0.72, 1.02)
Oh et al. [2015] [7]	Sweden	Cohort, Prospective	1992–2035	42,099	1395	30–76	Caffeinated	Premenopausal	RR	0.87 (0.7, 1.07)
Hashibe M. et al. [2015] [8]	USA	Cohort	1992–2011	50,563	1703	55–74	Caffeinated	Dose	RR	0.86 (0.73, 1.02)
Arthur R. et al. [2018] [9]	Canada	Cohort	7.0 (3.9–10.6)	3120	922	51 [44–63]	Caffeine	Premenopausal	HR	1.11 (0.82, 1.5)
Arthur R. et al. [2018] [9]	Canada	Cohort	7.0 (3.9–10.6)	3120	922	51 [44–63]	Caffeine	Postmenopausal	HR	1.01 (0.75, 1.35)
Zheng et al. [2021] [10]	USA	Cohort, Prospective	22	79,871	4719	63.4 [50–79]	Caffeinated	Caffeine dose & ER + PR+	HR	1.03 (0.92, 1.16)
Zheng et al. [2021] [10]	USA	Cohort, Prospective	22	79,871	4719	63.4[50–79]	Caffeinated	Caffeine dose & ER − PR−	HR	1.09 (0.84, 1.43)
Zheng et al. [2021] [10]	USA	Cohort, Prospective	22	79,871	4719	63.4 [50–79]	Caffeinated	Caffeine dose & ER − PR+	HR	1.05 (0.45, 2.51)
Zheng et al. [2021] [10]	USA	Cohort, Prospective	22	79,871	4719	63.4 [50–79]	Caffeinated	Caffeine dose & ER + PR−	HR	1.09 (0.83, 1.42)
Yaghjyan et al. [2022] [11]	USA	Cohort, Prospective	> 2–23.4	77,688	5005	63.8 [49–81]	Caffeinated	Caffeine dose & ER − PR−	HR	0.90 (0.73, 1.11)
Yaghjyan et al. [2022] [11]	USA	Cohort, Prospective	> 2–23.4	77,688	5005	63.8 [49–81]	Caffeinated	Caffeine dose & ER + PR+	HR	1.13 (1.03, 1.24)
**Decaffeinated coffee**										
Ishitani. K. et al. [2008] [12]	USA	Cohort, Retrospective	1992–2004	38,432	1188	≥ 45	Decaffeinated	Dose & Postmenopausal	RR	0.92 (0.74, 1.14)
Ishitani. K. et al. [2008] [4]	USA	Cohort, Retrospective	1992–2004	38,432	1188	≥ 45	Decaffeinated	Dose & Premenopausal	RR	1.05 (0.75, 1.49)
Boggs D. A. et al. [2010] [5]	USA	Cohort, Retrospective	1995–2007	52,062	1268	21–69	Decaffeinated	Dose & Postmenopausal	IRR	0.93 (0.65, 1.33)
Boggs D. A. et al. [2010] [5]	USA	Cohort, Retrospective	1995–2007	52,062	1268	21–69	Decaffeinated	Dose & Premenopausal	IRR	0.99 (0.56, 1.72)
Gierach G. L. et al. [2012] [12]	USA	Cohort	1995–2006	198,404	9915	50–71	Decaffeinated	Dose & ER − PR−	RR	1.13 (0.62, 2.05)
Gierach G. L. et al. [2012] [12]	USA	Cohort	1995–2006	198,404	9915	50–71	Decaffeinated	Dose & ER + PR−	RR	0.95 (0.49, 1.85)
Gierach G. L. et al. [2012] [12]	USA	Cohort	1995–2006	198,404	9915	50–71	Decaffeinated	Dose & ER + PR+	RR	1.18 (0.88, 1.57)
Bhoo‐Pathy N. et al. [2015] [13]	Europe	Cohort, Retrospective	1992–2010	335,060	10198	51 [25–70]	Decaffeinated	Dose & Postmenopausal	HR	0.97 (0.87, 1.08)
Bhoo‐Pathy N. et al. [2015] [13]	Europe	Cohort, Retrospective	1992–2010	335,060	10198	51 [25–70]	Decaffeinated	Dose & Premenopausal	HR	1.2 (0.9, 1.6)
Arthur R. et al. [2018] [9]	Canada	Cohort	7.0 (3.9–10.6)	3120	922	51 [44–63]	Decaffeinated	Dose & Postmenopausal	HR	0.99 (0.59, 1.65)
Arthur R. et al. [2018] [9]	Canada	Cohort	7.0 (3.9–10.6)	3120	922	51 [44–63]	Decaffeinated	Dose & Premenopausal	HR	0.85 (0.45, 1.6)
Gapstur S. M. et al. [2020] [12]	USA	Cohort, Prospective	2	57,075	2980	> 55	Decaffeinated	Dose & ER and PR−	HR	1.10 (0.78, 1.56)
Gapstur S. M. et al. [2020] [12]	USA	Cohort, Prospective	2	57,075	2980	> 55	Decaffeinated	Dose & ER and PR+	HR	1.08 (0.95, 1.22)
Farvid M. S. et al. [2021] [14]	USA	Cohort, Retrospective	30	8900	1054	30–55	Decaffeinated	Dose	HR	0.93 (0.75, 1.16)
Zheng et al. [2021] [10]	USA	Cohort, Retrospective	22	79,871	4719	63.4 [50–79]	Decaffeinated	Dose	HR	0.92 (0.72, 1.18)
**Caffeinated coffee**										
Vatten et al. [1990] (C) [15]	Norway	Cohort, Retrospective	1974–1985	14,593	152	35–51	Coffee	Dose	IRR	0.8 (0.5, 1.4)
Hoyer et al. [1992] [16]	Denmark	Cohort	1964–1986	5207	51	30–80	Coffee	Dose	RR	1.7 (0.7, 4.3)
Hunter et al. [1992] [17]	USA	Cohort	1980–1987	89,494	1439	30–55	Coffee	Dose	RR	0.90 (0.76, 1.04)
Folsom A. R. et al. [1993] [1]	USA	Cohort	1986–1990	34,388	580	62 ± 4 [55–69]	Coffee	Dose	RR	1.02 (0.79, 1.3)
TJ Key, et al. [1999] [18]	Japan	Cohort	1969–1993	34,759	344	< 40– ≥ 80	Coffee	Dose	RR	1.19 (0.93, 1.52)
Hirvonen et al, [2006] [19]	France	Cohort	1994–2002	4396	95	47.16 ± 6.6 [35–60]	Coffee (caffeinated + decaffeinated)	Dose	RR	1.10 (0.66, 1.84)
Nkondjock et al. [2006] [20]	USA, Canada, Poland and Israel	Cohort, Retrospective	1977–2002	1690	845	≤ 64	Caffeinated Coffee	Dose	OR	0.31 (0.13, 0.71)
Ishitani. K. et al. [2008] [4]	USA	Cohort, Retrospective	1992–2004	38,432	1188	≥ 45	Caffeinated Coffee	Dose & Postmenopausal	RR	1.08 (0.85, 1.38)
Ishitani. K. et al. [2008] [4]	USA	Cohort, Retrospective	1992–2004	38,432	1188	≥ 45	Caffeinated Coffee	Dose & Premenopausal	RR	0.97 (0.64, 1.46)
Ganmaa, D. et al. [2008] [3]	USA	Cohort, Retrospective	1980–2002	85,987	5272	30–55	Coffee	Dose	RR	0.92 (0.82, 1.03)
Bissonauth, V., et al. [2009] [21]	Canada	Nested case‐control	2004–2006	560	280	50	Caffeinated Coffee	Dose & Postmenopausal	OR	1.3 (0.66, 1.88)
Bissonauth, V., et al. [2009] [21]	Canada	Nested case‐control	2004–2006	560	280	50	Caffeinated Coffee	Dose & Premenopausal	OR	1.09 (0.45, 1.99)
Larsson S. C. et al. [2009] [22]	Sweden	Cohort, Prospective	1990–2007	61,433	2952	40–76	Coffee	Dose & ER − PR−	RR	0.91 (0.59, 1.38)
Larsson S. C. et al. [2009] [22]	Sweden	Cohort, Prospective	1990–2007	61,433	2952	40–76	Coffee	Dose & ER + PR−	RR	0.98 (0.63, 1.53)
Larsson S. C. et al. [2009] [22]	Sweden	Cohort, Prospective	1990–2007	61,433	2952	40–76	Coffee	Dose & ER + PR+	RR	1.12 (0.87, 1.44)
Nilsson et al, [2010] [23]	Sweden	Cohort, Prospective	1992–2007	64,603	3034	24.1 (median)	NR	Dose	HR	0.92 (0.68, 1.25)
Bhoo Pathy N. et al. [2010] [20]	Netherland	Cohort, Retrospective	1993–1997	27,323	10,198	52.6 [20–70]	Coffee	Dose & Postmenopausal	HR	1.09 (0.71, 1.68)
Bhoo Pathy N. et al. [2010] [20]	Netherland	Cohort, Retrospective	1993–1997	27,323	10,198	52.6 [20–70]	Coffee	Dose & Premenopausal	HR	0.84(0.59, 1.2)
Boggs D. A. et al. [2010] [5]	USA	Cohort, Retrospective	1995–2007	52,062	1268	21–69	Caffeinated	Dose & Postmenopausal & ER − PR−	IRR	1.00 (0.56, 1.79)
Boggs D. A. et al. [2010] [5]	USA	Cohort, Retrospective	1995–2007	52,062	1268	21–69	Caffeinated	Dose & Premenopausal & ER − PR−	IRR	1.10 (0.6, 2.02)
Boggs D. A. et al. [2010] [5]	USA	Cohort, Retrospective	1995–2007	52,062	1268	21–69	Caffeinated	Dose & Postmenopausal & ER + PR+	IRR	0.89 (0.55, 1.43)
Boggs D. A. et al. [2010] [5]	USA	Cohort, Retrospective	1995–2007	52,062	1268	21–69	Caffeinated	Dose & Premenopausal & ER + PR+	IRR	1.14 (0.67, 1.94)
Fagherazzi. G. et al. [2011] [6]	France	Cohort, Retrospective	1993–2005	67,703	2868	40–65	Regular or decaffeinated	Dose & ER − PR−	HR	0.81 (0.61, 1.07)
Fagherazzi. G. et al. [2011] [6]	France	Cohort, Retrospective	1993–2005	67,703	2868	40–65	Regular or decaffeinated	Dose & ER − PR+	HR	0.66 (0.39, 1.12)
Fagherazzi. G. et al. [2011] [6]	France	Cohort, Retrospective	1993–2005	67,703	2868	40–65	Regular or decaffeinated	Dose & ER + PR−	HR	1.05 (0.83, 1.34)
Fagherazzi. G. et al. [2011] [6]	France	Cohort, Retrospective	1993–2005	67,703	2868	40–65	Regular or decaffeinated	Dose & ER + PR+	HR	0.98 (0.85, 1.2)
Gierach G. L. et al. [2012] [24]	USA	Cohort	1995–2006	198,404	9915	50–71	Caffeinated	Dose & ER − PR−	RR	1.09 (0.72, 1.65)
Gierach G. L. et al. [2012] [24]	USA	Cohort	1995–2006	198,404	9915	50–71	Caffeinated	Dose & ER − PR+	RR	1.38 (0.39, 4.88)
Gierach G. L. et al. [2012] [24]	USA	Cohort	1995–2006	198,404	9915	50–71	Caffeinated	Dose & ER + PR−	RR	0.98 (0.62, 1.55)
Gierach G. L. et al. [2012] [24]	USA	Cohort	1995‐2006	198,404	9915	50–71	Caffeinated	Dose & ER + PR+	RR	1.09 (0.88, 1.34)
Harris H. R. et al. [2012] [25]	Sweden	Cohort	1987–2010	3243	394	52.25	Most caffeinated Coffee	Dose	HR	1.14 (0.71, 1.83)
Hashibe M. et al. [2015] [8]	USA	Cohort	1992–2011	50,563	1703	55–74	Total (caffeinated and decaffeinated)	Dose	RR	0.97 (0.87, 1.08)
Bhoo‐Pathy N. et al. [2015] [13]	Europe	Cohort, Retrospective	1992–2010	335,060	681	51 [25–70]	Caffeinated	Dose & 25 ≥ BMI	HR	0.90 (0.82, 0.98)
Bhoo‐Pathy N. et al. [2015] [13]	Europe	Cohort, Retrospective	1992–2010	335,060	681	51 [25–70]	Caffeinated	Dose & 25 < BMI	HR	1.19 (0.93, 1.53)
Oh et al. [2015] [7]	Sweden	Cohort, Prospective	1992–2037	42,099	1395	30–66	Caffeinated	Dose & Postmenopausal	RR	0.81 (0.67, 0.97)
Oh et al. [2015] [7]	Sweden	Cohort, Prospective	1992–2037	42,099	1395	30–66	Caffeinated	Dose & Premenopausal	RR	0.82 (0.65, 1.03)
Arthur R. et al. [2018] [9]	Canada	Cohort	7.0 (3.9–10.6)	3120	922	51 [44–63]	Caffeinated	Dose & Postmenopausal	HR	0.82 (0.45, 1.49)
Yaghjyan et al. [2018] [26]	United Kingdom	Cohort, Prospective	5	126,182	2636	40–69	Coffee	Dose & Postmenopausal	HR	0.98 (0.87, 1.1)
Sánchez‐Quesada et al. [2020] [27]	Spain	Cohort, Retrospective	19	10,812	101	32	Total (caffeinated + decaffeinated)	Dose & Premenopausal	HR	1.69 (0.96, 2.96)
Sánchez‐Quesada et al. [2020] [27]	Spain	Cohort, Retrospective	19	10,812	101	32	Total (caffeinated + decaffeinated)	Dose & Postmenopausal	HR	0.44 (0.21, 0.92)
Schmit et al. [2020] [28]	USA	Nested case–control	2002–2009	3795	458	55.3	Regular coffee + decaffeinated coffee	Dose	OR	0.8 (0.48, 1.33)
Gapstur S. M. et al. [2020] [12]	USA	Cohort, Prospective	2	57,075	2980	> 55	Caffeinated	Dose & ER and PR−	HR	0.97 (0.71, 1.32)
Gapstur S. M. et al. [2020] [12]	USA	Cohort, Prospective	2	57,075	2980	> 55	Caffeinated	Dose & ER or PR+	HR	0.93 (0.83, 1.03)
Sinnadurai et al. [2020] [29]	Japan	Cohort, Prospective	20	33,396	255	57.7 [40–79]	Coffee	Dose	OR	0.84 (0.64, 1.1)
Zheng et al. [2021] [10]	USA	Cohort, Prospective	22	79,871	4719	63.4 [50–79]	Caffeinated	Dose & Postmenopausal	HR	1.02 (0.88, 1.18)
Farvid M. S. et al. [2021] [14]	USA	Cohort, Prospective	30	8900	1054	30–55	Regular Coffee	Dose	HR	0.57 (0.41, 0.78)
Yaghjyan et al. [2022] [26]	USA	Cohort, Prospective	> 2–23.4	77,688	5005	63.8 [49–81]	Caffeinated Coffee	Dose & ER + PR+ & Postmenopausal	HR	1.09 (0.61, 1.96)
Yaghjyan et al. [2022] [26]	USA	Cohort, Prospective	> 2–23.4	77,688	5005	63.8 [49–81]	Caffeinated Coffee	Dose & ER‐PR− & Postmenopausal	HR	1.10 (0.86, 1.41)
Lin et al. [2025] [30]	China	Cohort, Prospective	2003–2020	13,567	285	61.92 ± 6.63	Total (caffeinated and decaffeinated)	Dose	HR	1.19 (0.65, 2.54)

Abbreviations: HR, hazard ratio; NR, none reported; OR, odds ratio; RR, risk ratio.

A total of 11 cohort studies [29, 30, 31, 32, 33, 34, 35, 36, 37, 38, 39] evaluated the association between caffeine intake and breast cancer risk. These studies were predominantly prospective in design and were conducted across North America and Europe, with large sample sizes ranging from approximately 3000 to over 85,000 participants and follow‐up periods extending up to more than two decades. Several studies provided stratified analyzes according to menopausal status or hormone receptor subtypes (estrogen receptor [ER] and progesterone receptor [PR] status). Overall, the included studies demonstrated substantial methodological consistency, with multivariable adjustment for key confounders, including age, body mass index, reproductive factors, and lifestyle behaviors.

Seven cohort studies [20, 32, 33, 37, 38, 40, 41] investigated the association between decaffeinated coffee consumption and breast cancer risk. These studies were conducted primarily in the United States, Europe, and Canada and included both prospective and retrospective cohort designs. Sample sizes ranged widely, from approximately 3000 to over 335,000 participants, and follow‐up durations ranged from 2 to 30 years. Exposure assessment focused on habitual decaffeinated coffee intake, typically categorized by dose or consumption frequency. Several studies reported results stratified by menopausal status and hormone receptor subtypes. Effect estimates were generally reported as HRs, RRs, or IRRs, and most studies adjusted for a comprehensive set of dietary and non‐dietary confounders.

Caffeinated coffee consumption was examined in 30 studies [20, 29, 31, 32, 33, 34, 35, 36, 37, 38, 40, 41, 42, 43, 44, 45, 46, 47, 48], making it the most extensively studied exposure among the included analyzes. These studies encompassed diverse geographic regions, including North America, Europe, and Asia, and featured a wide range of cohort sizes, from fewer than 1000 participants in nested case–control analyzes to more than 198,000 participants in large prospective cohorts. Follow‐up periods ranged from approximately 5 to over 30 years. Most studies assessed caffeinated coffee intake quantitatively and reported associations using RRs, HRs, ORs, or IRRs, with many providing subgroup analyzes by menopausal status, hormone receptor status, or body mass index. Overall, the studies demonstrated heterogeneity in exposure definitions but were generally of moderate to high methodological quality, with extensive adjustment for potential confounders.

### 3.3 Quality Assessment

Using the Newcastle–Ottawa Scale, 28 of the 31 included cohort studies were classified as high quality (7–9 stars), whereas three studies were of moderate quality (5–6 stars). No study was rated as low quality. Most studies achieved full scores in the outcome domain due to registry‐based outcome ascertainment and adequate follow‐up duration. Moderate ratings were primarily driven by limited comparability due to restricted confounder adjustment or highly selected populations (Table 2).

**Table 2:** Quality assessment of included cohort studies using the Newcastle–Ottawa Scale (NOS).

Study	Selection (max 4 ★)	Comparability (max 2 ★)	Outcome (max 3 ★)	Total (★/9)	Quality
Vatten et al. 1990	★★★✩	★✩	★★★	**7**	High
Høyer et al. 1992	★★★★	★✩	★★★	**8**	High
Hunter et al.1992	★★✩✩	★✩	★★★	**6**	Moderate
Folsom et al.1993	★★★✩	★★	★★✩	**7**	High
Michels et al. 2002	★★★✩	★★	★★★	**8**	High
Hirvonen et al. 2006	★★★✩	★✩	★★★	**7**	High
Nkondjock et al. 2006	★★✩✩	★✩	★★★	**6**	Moderate
Ganmaa et al. 2008	★★✩✩	★★	★★★	**7**	High
Ishitani et al. 2008	★★✩✩	★★	★★★	**7**	High
Bissonauth et al. 2009	★★✩✩	★✩	★★✩	**5**	Moderate
Larsson et al. 2009	★★★✩	★★	★★★	**8**	High
Boggs et al. 2010	★★✩✩	★★	★★★	**7**	High
Nilsson et al. 2010	★★★✩	★★	★★★	**8**	High
Bhoo‐Pathy et al. 2010	★★★✩	★★	★★★	**8**	High
Fagherazzi et al. 2011	★★✩✩	★★	★★★	**7**	High
Gierach et al. 2012	★★★✩	★★	★★★	**8**	High
Harris et al. 2012	★★★✩	★★	★★★	**8**	High
Hashibe et al. 2015	★★✩✩	★★	★★★	**7**	High
Oh et al. 2015	★★★✩	★★	★★★	**8**	High
Bhoo‐Pathy et al. 2015	★★★✩	★★	★★★	**8**	High
Arthur et al. 2018	★★✩✩	★★	★★★	**7**	High
Yaghjyan et al. 2018	★★✩✩	★★	★★★	**7**	High
Gapstur et al. 2020	★★✩✩	★★	★★★	**7**	High
Sánchez‐Quesada et al. 2020	★★✩✩	★★	★★★	**7**	High
Schmit et al. 2020	★★★✩	★★	★★★	**8**	High
Sinnadurai et al. 2020	★★★✩	★★	★★★	**8**	High
Zheng et al. 2021	★★✩✩	★★	★★★	**7**	High
Farvid et al. 2021	★★✩✩	★★	★★★	**7**	High
Yaghjyan et al. 2022	★★✩✩	★★	★★★	**7**	High
Lin et al. 2025	★★★✩	★★	★★★	**8**	High

### 3.4 Meta‐Analysis: Caffeine and Breast Cancer Risk

**Figure 2:**
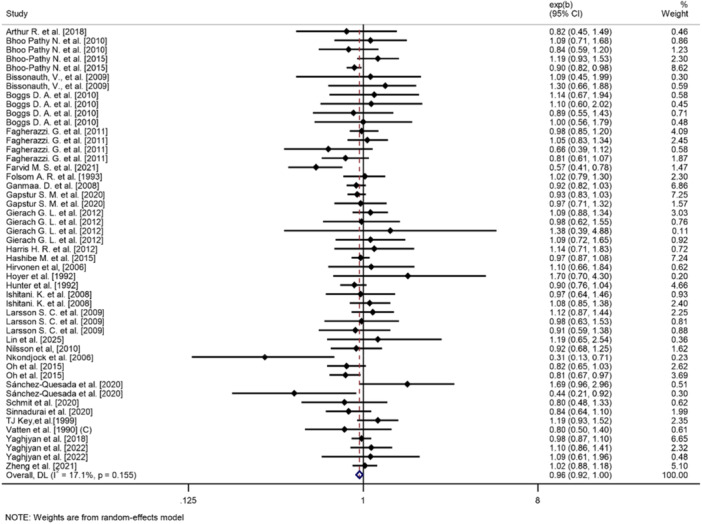
Forest plot demonstrating the overall hazard ratio (HR) with 95% confidence interval (CI) for the association between regular coffee (caffeinated) consumption and risk of breast cancer in females.

The pooled analysis of 25 studies assessing caffeine intake and breast cancer risk showed no significant association between caffeine consumption and breast cancer among women (pooled HR = 0.99; 95% CI: 0.94–1.04; *p* = 0.82). Heterogeneity across studies was moderate (I^2^ = 36.0%, *p* = 0.04), suggesting some variability in effect estimates among the included studies. The forest plot (Figure 2) demonstrated that most studies clustered around the null value, indicating consistent findings across the included cohorts. These results suggest that overall caffeine intake is unlikely to exert a protective or harmful effect on breast cancer risk.

Subgroup analyzes of caffeine intake revealed similar, non‐significant associations across most categories (Table 3). When stratified by cohort duration, neither shorter (< 12 years) nor longer (≥ 12 years) follow‐up durations were associated with a significant difference. Likewise, no meaningful associations were observed across menopausal status (premenopausal vs. postmenopausal). Stratification by ER and PR status also yielded non‐significant findings, although a modest inverse trend was observed for ER‐/PR+ tumors (HR = 0.72; 95% CI: 0.47–1.09). Overall, the subgroup results reinforced the robustness of the null association between caffeine intake and breast cancer risk.

**Table 3:** Overall and subgroup meta‐analyzes findings for the association between coffee and caffeine intake and risk of breast cancer in adult women.

	Number of studies	WMD (95%CI)	*p*‐value	Heterogeneity	
				P heterogeneity	I^2^
**Caffeine intake and breast cancer risk**					
Overall effect size	25	0.99 (0.94, 1.04)	0.824	36.0%	0.039
Cohort duration					
< 12	10	1.03 (0.93, 1.14)	0.458	0.106	37.9%
≥ 12	15	0.97 (0.91, 1.03)	0.383	0.091	34.7%
Estrogen receptor					
ER−/PR−	6	1.00 (0.88, 1.13)	0.990	0.263	22.7%
ER + /PR+	6	0.98 (0.89, 1.07)	0.665	0.020	62.8%
ER−/PR+	3	0.72 (0.47, 1.09)	0.126	0.603	0.0%
ER + /PR−	4	1.03 (0.77, 1.36)	0.839	0.013	72.1%
Menopause status					
Postmenopausal	6	0.99 (0.89, 1.11)	0.957	0.090	47.6%
Premenopausal	3	0.95 (0.82, 1.10)	0.498	0.426	0.0%
**Regular coffee consumption and breast cancer risk**					
Overall effect size	49	0.95 (0.91, 0.99)	0.035*	0.155	17.1%
Cohort duration					
< 12	27	0.96 (0.91, 1.02)	0.214	0.220	16.7%
≥ 12	21	0.94 (0.88, 1.01)	0.108	0.160	23.6%
Estrogen receptor					
ER−/PR−	8	0.96 (0.83, 1.11)	0.615	0.900	0.0%
ER + /PR+	8	0.98 (0.91, 1.05)	0.566	0.679	0.0%
ER−/PR+	8	0.71 (0.45, 1.11)	0.136	0.546	0.0%
ER + /PR−	3	0.85 (0.60, 1.19)	0.358	0.017	70.4%
Menopause status					
Postmenopausal	14	0.94 (0.90, 0.99)	0.030*	0.672	0.0%
Premenopausal	6	0.99 (0.86, 1.15)	0.963	0.400	2.5%
**Decaffeinated coffee consumption and breast cancer risk**					
Overall effect size	15	1.00 (0.94, 1.07)	0.793	0.939	0.0%
Cohort duration					
< 12	9	1.02 (0.96, 1.10)	0.414	0.756	0.0%
≥ 12	6	0.93 (0.81, 1.06)	0.297	0.999	0.0%
Estrogen receptor					
ER−/PR−	2	1.10 (0.82, 1.49)	0.505	0.939	0.0%
ER + /PR+	2	1.09 (0.97, 1.22)	0.121	0.582	0.0%
ER + /PR−	1	0.95 (0.48, 1.84)	0.880	—	—
Menopause status					
Postmenopausal	7	0.99 (0.93, 1.06)	0.930	0.782	0.0%
Premenopausal	4	1.08 (0.89, 1.32)	0.404	0.758	0.0%

*Note:* Asterisk (*) indicates statistical significance (*p* < 0.05).

Abbreviations: CI, confidence interval; WMD, weighted mean differences.

### 3.5 Meta‐Analysis: Regular Coffee and Breast Cancer Risk

**Figure 3:**
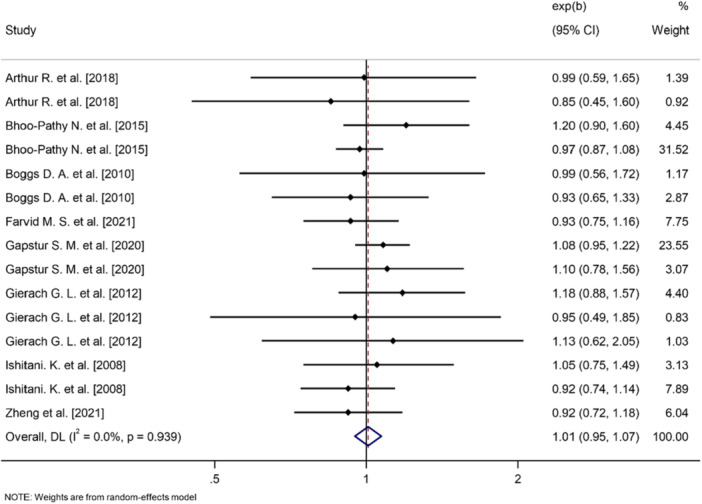
Forest plot demonstrating the overall hazard ratio (HR) with 95% confidence interval (CI) for the association between decaffeinated coffee consumption and risk of breast cancer in females.

A total of 31 independent cohort studies contributed 49 effect estimates to the meta‐analysis investigating regular coffee intake, which revealed a modest but statistically significant inverse association with breast cancer risk (pooled HR = 0.95; 95% CI: 0.91–0.99; *p* = 0.035). Heterogeneity among studies was low (I^2^ = 17.1%, *p* = 0.155), indicating good consistency across findings. The forest plot (Figure 3) indicated that most studies reported a slight reduction in risk among higher coffee consumers. These findings suggest that habitual coffee consumption may confer a small, modest inverse association against breast cancer in women.

Subgroup analyzes for regular coffee consumption further supported this trend in certain populations (Table 3). Among postmenopausal women, a statistically significant, modest inverse association was observed (HR = 0.94; 95% CI: 0.90–0.99; *p* = 0.03), whereas no association was observed among premenopausal women. Stratified analyzes by ER/PR status revealed no significant associations, although a stronger inverse association was observed for ER + /PR– tumors. Differences between short (< 12 years) and long (≥ 12 years) cohort durations were minimal, indicating that the modest inverse association persisted across study lengths. Overall, these results suggest a slight reduction in breast cancer risk with regular coffee intake, particularly among postmenopausal women (Table 3).

### 3.6 Meta‐Analysis: Decaffeinated Coffee and Breast Cancer Risk

**Figure 4:**
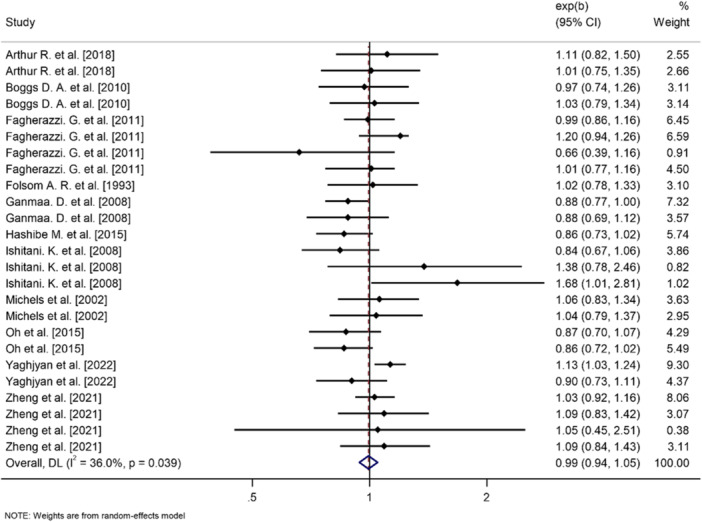
Forest plot demonstrating the overall hazard ratio (HR) with 95% confidence interval (CI) for the association between caffeine intake and risk of breast cancer in females.

In contrast, the pooled results from 15 studies evaluating decaffeinated coffee consumption showed no significant relationship with breast cancer risk (HR = 1.00; 95% CI: 0.94–1.07; *p* = 0.793). There was no heterogeneity across studies (I^2^ = 0.0%, *p* = 0.94), reflecting highly consistent findings (Figure 4).

Subgroup analyzes by cohort duration, menopausal status, and hormone receptor subtype did not alter the overall null findings. No significant associations were observed for shorter (< 12 years) or longer (≥ 12 years) follow‐ups. Similarly, both premenopausal and postmenopausal subgroups showed no significant effects. Across ER/PR subtypes, all results were null, suggesting that the absence of association is consistent across different hormonal and demographic contexts. Thus, the findings collectively indicate that decaffeinated coffee has no measurable impact on breast cancer risk in women (Table 3).

### 3.7 Sensitivity Analysis

Sensitivity analyzes were performed to evaluate the robustness of the pooled estimates for the associations between caffeine, caffeinated coffee, and decaffeinated coffee intake and breast cancer risk. Sequential leave‐one‐out analyzes showed that the overall effect estimates were not materially altered by the exclusion of any single study, indicating that no individual study disproportionately influenced the results.

### 3.8 Publication Bias

**Figure 5:**
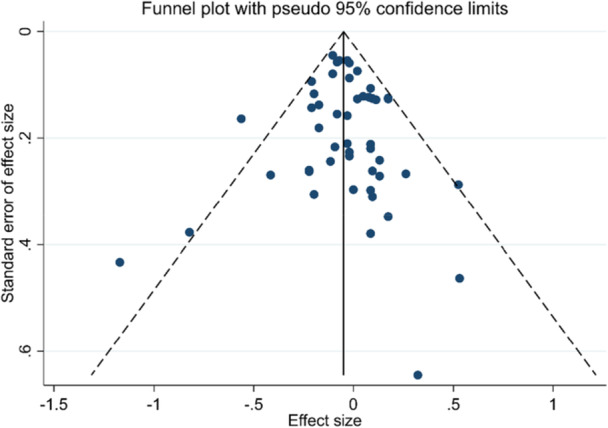
Funnel plot demonstrating the publication bias for the association between regular coffee (caffeinated) consumption and risk of breast cancer in females.

**Figure 6:**
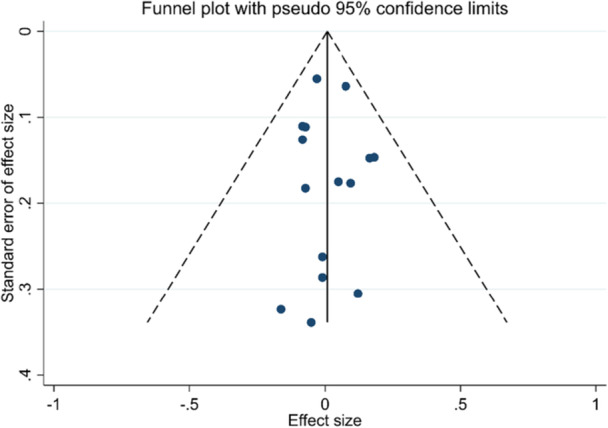
Funnel plot demonstrating the publication bias for the association between decaffeinated coffee consumption and risk of breast cancer in females.

**Figure 7:**
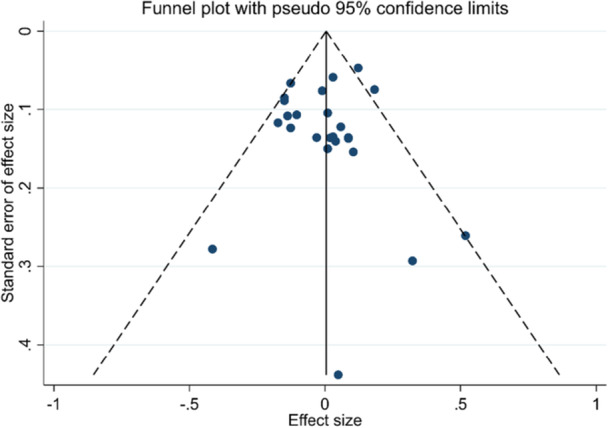
Funnel plot demonstrating the publication bias for the association between caffeine intake and risk of breast cancer in females.

Publication bias was evaluated using funnel plots and assessed statistically using Begg's and Egger's tests (Table 4; Figures 5, 6, 7). The funnel plots for regular, decaffeinated, and caffeine intake studies appeared symmetrical, suggesting no significant evidence of publication bias. This finding was further supported by non‐significant Begg's and Egger's test results for all exposures (Begg's test *p* = 0.776, 0.767, and 0.199; Egger's test *p* = 0.609, 0.908, and 0.687 for regular coffee, decaffeinated coffee, and caffeine intake, respectively). These results indicate that the overall meta‐analysis findings were unlikely to be influenced by publication bias.

**Table 4:** Publication bias and sensitivity analysis.

Outcomes	Publication bias		Sensitivity analysis
	Begg's Test	Egger's test	
Caffeine and breast cancer risk	0.199	0.687	None
Regular coffee and breast cancer risk	0.776	0.609	None
Decaffeinated coffee and breast cancer risk	0.767	0.908	None

## 4 Discussion

### 4.1 Aim and Findings

This meta‐analysis incorporated 49 cohort studies, and the pooled findings revealed that regular coffee consumption was modestly associated with a lower risk of breast cancer. In contrast, neither decaffeinated coffee nor total caffeine intake showed any significant association with breast cancer risk. Subgroup analyzes provided further insight into the observed associations. Stratification by menopausal status revealed a significant inverse relationship among postmenopausal women, with regular coffee consumption associated with a modest reduction in breast cancer risk. No meaningful association was observed among premenopausal women. Consistently, neither decaffeinated coffee nor total caffeine intake showed any association with breast cancer risk within either menopausal subgroup. When studies were stratified by cohort duration, the inverse association between regular coffee intake and breast cancer persisted across both short‐term (< 12 years) and long‐term (≥ 12 years) follow‐up periods. However, neither subgroup reached statistical significance independently. For both caffeinated and decaffeinated coffee, the results were stable across durations, indicating no association with study length. Analyzes by ER and PR status also demonstrated no statistically significant associations. Regular coffee intake yielded similar risk estimates across ER + /PR + , ER − /PR − , ER + /PR − , and ER − /PR+ subtypes, with a non‐significant tendency toward reduced risk observed for ER − /PR+ tumors. Likewise, caffeine and decaffeinated coffee showed null results across receptor‐defined subgroups.

### 4.2 Scientific Literature

Previous investigations have consistently aligned with our findings. A meta‐analysis by Tang et al. [59] identified a slight, although statistically nonsignificant, reduction in breast cancer risk among regular coffee drinkers, consistent in direction with our pooled estimates. Expanding upon this evidence, Li et al. [22] conducted a more extensive synthesis encompassing 26 observational studies and found that higher coffee intake was associated with a reduced risk of breast cancer, particularly among postmenopausal women. This pattern closely mirrors our subgroup analysis, in which the modest inverse association was similarly confined to postmenopausal populations. The most recent and methodologically rigorous meta‐analysis by Li and Ma [60] further reinforced these trends, demonstrating a statistically significant inverse association with total coffee intake among postmenopausal women, whereas no meaningful associations were found for premenopausal women, caffeine, or decaffeinated coffee. These findings are fully concordant with the present analysis.

Several large cohort investigations further support this pattern. Nkondjock et al. [47] reported a striking 69% risk reduction among BRCA1/2 mutation carriers consuming six or more cups of coffee daily. Similarly, Sánchez‐Quesada et al. [55] reported a 56% lower risk of postmenopausal breast cancer with higher total coffee intake. This finding aligns with our subgroup results and the evidence from the European Prospective Investigation into Nutrition and Cancer (EPIC) cohort [20], which included 335,060 women, as well as other studies in this context [21]. Consistent evidence also arises from Kim et al. [61], whose cross‐sectional analysis of 162,000 Korean adults found a 44% lower risk among high coffee consumers, underscoring the reproducibility of this association across diverse populations. The Women's Health Initiative (WHI) study by Zheng et al. [38] reported no significant association between caffeine intake and breast cancer risk, a finding consistent with our null results for caffeine exposure.

Conversely, several cohorts have reported findings that diverge from ours. Sinnadurai et al. [57] and Gapstur et al. [40] observed no significant association between coffee consumption and breast cancer, which may reflect variations in intake patterns, bean composition, or residual confounding from lifestyle factors such as alcohol use and smoking. Schmit et al. [56] likewise found no association among African American women, a population characterized by generally low consumption levels. A subsequent WHI analysis by Yaghjyan et al. [39] identified a modest positive association with ER + /PR+ tumors, while another investigation [32] reported a higher risk of ER − /PR− breast cancer among women with greater caffeine consumption. These findings point to potential heterogeneity in the relationship between caffeine and breast cancer across hormone receptor subtypes. The observed divergence may arise from variations in population characteristics, exposure assessment, or adjustment for hormonal and behavioral confounders. Biologically, such differences could reflect receptor‐specific mechanisms, including variations in hormonal responsiveness, metabolic activation pathways, or interactions with estrogen signaling, underscoring the complexity of caffeine's role in breast carcinogenesis.

### 4.3 Mechanism of Action

Coffee and its bioactive constituents interact with multiple molecular pathways that converge on key mechanisms of breast cancer pathophysiology. Caffeine and related methylxanthines modulate estrogen metabolism; higher coffee and caffeine intake have been associated with increased urinary 2‐hydroxyestrone and altered ratios of estrogen metabolites in premenopausal women, reflecting the induction of hepatic cytochrome P450 enzymes, particularly CYP1A2, which catalyze estrogen hydroxylation [61, 62]. Coffee intake also appears to interact with caffeine‐metabolizing genetic variants, such as CYP1A2*1 F, influencing breast volume, a surrogate of cumulative estrogen exposure, and suggesting that metabolic genotype modifies hormonally mediated breast tissue responses [63]. These findings position the hormonal axis, enhanced estrogen clearance, elevated sex hormone‐binding globulin (SHBG), and reduced circulating free estradiol—as a primary mechanism through which coffee may attenuate breast cancer risk.

Beyond hormonal modulation, coffee's non‐caffeine bioactive compounds (such as chlorogenic and caffeic acids, kahweol, cafestol, trigonelline, and enterolactone precursors) exert complementary antioxidant, anti‐inflammatory, and pro‐apoptotic effects [63, 64, 65]. Chlorogenic acid (CGA), a major coffee polyphenol, inhibits NF‐κB signaling and suppresses breast cancer cell migration, invasion, and proliferation, while promoting mitochondrial apoptosis via upregulation of p53 and Bax, downregulation of Bcl‐2, and activation of caspase‐3 [66, 67]. Similarly, kahweol and cafestol downregulate Sp1, survivin, and Mcl‐1 while activating caspase‐3, thereby restricting tumor cell survival and growth [65]. Caffeine itself contributes distinct tumor‐suppressive effects by antagonizing adenosine A2A/A2B receptors, which induce p16, p21, p53, and caveolin‐1 expression while inhibiting oncogenic Akt and Erk1/2 pathways in breast cancer–associated fibroblasts [69]. Furthermore, higher coffee intake has been linked with reduced insulin‐like growth factor‐1 receptor (IGF1R) expression in breast tumors, particularly among normal‐weight women, indicating attenuation of insulin/IGF signaling, another axis implicated in tumor proliferation and survival [70]. These mechanisms converge to reduce oxidative and inflammatory stress, suppress proliferative signaling, enhance xenobiotic detoxification, and promote programmed cell death in mammary tissue.

Epigenetic regulation and ligand‐sensing mechanisms represent additional routes through which coffee may influence cancer biology. Experimental evidence suggests that coffee constituents can remodel the landscapes of DNA methylation and histone modifications, with caffeic and chlorogenic acids shown to restrict hypermethylation at tumor‐suppressor loci, such as RARβ [71]. Broader antioxidant effects may further normalize aberrant methylation patterns implicated in breast cancer progression. In parallel, diet‐derived ligands in coffee can activate the aryl hydrocarbon receptor (AhR), a context‐dependent modulator of tumor behavior, and stimulate AhR/NRF2‐linked transcriptional responses in intestinal epithelia, raising the possibility of systemic, ligand‐driven cross‐talk that extends to mammary tissue [71, 72, 73]. These epigenetic and receptor‐mediated effects complement the redox, inflammatory, metabolic, and DNA‐damage–response mechanisms previously described, outlining a multifaceted network through which coffee's bioactive compounds may modulate breast cancer pathophysiology.

### 4.4 Clinical Implications

The findings of this meta‐analysis indicate that moderate coffee consumption can be safely included in lifestyle recommendations, as it is associated with a modest reduction in breast cancer risk, particularly among postmenopausal women, without evidence of harm. Clinically, this suggests that routine restriction of coffee intake solely to reduce breast cancer risk is unnecessary. While specific quantitative intake guidelines cannot be derived from the current evidence, recommendations should be individualized, considering factors such as genetic variability in caffeine metabolism, estrogen pathways, and comorbidities like anxiety or cardiovascular disease. At the population level, coffee may be recognized as a widely accepted, low‐cost beverage with potential multisystem benefits, and could be incorporated into broader dietary guidance as a complementary component within established cancer prevention and healthy eating strategies.

### 4.5 Strengths and Limitations

This meta‐analysis demonstrates several notable strengths that enhance the validity and reliability of its conclusions. It is among the most comprehensive and current quantitative syntheses examining total, caffeinated, and decaffeinated coffee intake in relation to breast cancer risk, incorporating numerous diverse, high‐quality prospective cohort studies. By applying standardized exposure definitions, extensive multivariable adjustments, rigorous risk‐of‐bias assessments using ROBINS‐I, and adherence to PRISMA 2020 guidelines, the study minimizes heterogeneity, recall bias, and confounding compared with earlier analyzes. Stratified analyzes by menopausal status, hormone receptor subtype, and follow‐up duration further strengthen biological plausibility and interpretive robustness. However, certain limitations inherent to observational meta‐analyzes remain. Residual confounding, self‐reported exposure measures, and variability in coffee composition and preparation methods may still affect precision. Limited data on hormone therapy use, reproductive factors, and rarer tumor subtypes reduce power for some subgroup analyzes. The study protocol was not registered in PROSPERO. Additionally, small‐study effects, publication bias, and inter‐study variation in diagnostic criteria and follow‐up duration cannot be entirely excluded. Despite these caveats, the study's methodological rigor and comprehensive scope provide a reliable and nuanced synthesis of the current evidence on the link between coffee consumption and breast cancer risk.

### 4.6 Future Research Directions

Future research on coffee and breast cancer should move beyond simple associations toward understanding causal mechanisms, focusing on dose–response relationships, bioactive compounds, and their effects on pathways like oxidative stress, estrogen metabolism, and inflammation. Studies should consider genetic variability, tumor subtypes, timing, duration, and preparation methods of coffee consumption, using biomarkers and multi‐omics approaches to improve accuracy. Investigating interactions with lifestyle factors and including diverse populations will enhance generalizability. Integrating molecular, epidemiologic, and personalized approaches will clarify not only whether coffee influences breast cancer risk, but also how, in whom, and under what conditions, supporting evidence‐based, individualized dietary recommendations.

## 5 Conclusion

In conclusion, this meta‐analysis offers coherent and reproducible evidence that habitual coffee consumption is linked to a modest yet potentially meaningful reduction in breast cancer risk, most notably among postmenopausal women. The absence of a significant association with both caffeine and decaffeinated coffee suggests that the modest inverse association may arise from other bioactive compounds in coffee that modulate carcinogenic processes through anti‐inflammatory, antioxidant, and anti‐proliferative mechanisms. These results point to a plausible chemopreventive role of coffee in breast cancer, while emphasizing that causality cannot yet be inferred. Future investigations should focus on prospectively designed cohorts and mechanistic studies to delineate the biological pathways involved, characterize dose–response relationships, and determine whether specific hormonal‐ or receptor‐defined subgroups experience differential benefits from coffee consumption.

## Author Contributions

**Mehdi Karimi:** conceptualization, funding acquisition, investigation, writing – original draft, writing – review and editing, validation, visualization, methodology, software, project administration, formal analysis, resources, supervision, data curation. **Shadi Ghaemi:** data curation, supervision, resources, project administration, software, methodology, validation, funding acquisition, investigation. **Razieh Goudarzi:** data curation, supervision, resources, software, project administration, methodology, validation, investigation, funding acquisition. **Mohammad Amin Karimi:** writing – original draft, writing – review and editing, conceptualization, validation, methodology, project administration, resources, investigation. **Kimia Kazemi:** methodology, conceptualization, writing – original draft, investigation, validation. **Hoda Haghshenas:** writing – original draft, investigation, conceptualization, validation, visualization. **Omid Asbaghi:** project administration, supervision, resources, formal analysis, data curation, software, methodology, validation, investigation, conceptualization.

## Funding

The authors have nothing to report.

## Ethics Statement

The study is based on previously published data and does not involve new human or animal experimentation.

## Conflicts of Interest

The authors declare no conflicts of interest.

## Transparency Statement

The lead author Mehdi Karimi, Omid Asbaghi affirms that this article is an honest, accurate, and transparent account of the study being reported; that no important aspects of the study have been omitted; and that any discrepancies from the study as planned (and, if relevant, registered) have been explained.

## Supporting information

Supporting File

## Data Availability

The data that support the findings of this study are available from the corresponding author upon reasonable request.
