# Supplementary material for: Association Between Coffee and Caffeine Intake and Risk of Breast Cancer: A Systematic Review and Meta‐Analysis of Cohort Studies
Source: Health Sci Rep. 2026 Jun 3;9(6):e72580. doi: 10.1002/hsr2.72580 (PMC13282461; doi:10.1002/hsr2.72580)
Supplement: Supplementary file 1 — Supporting File [file HSR2-9-e72580-s001.docx]

- Title:

**Association Between Coffee and Caffeine** **Intake and Risk of Breast Cancer: A Systematic Review and Meta-Analysis of Cohort Studies**

- First author: Mehdi Karimi (*Karimi M. et al. 2025*)

**Supplementary Materials**

**Supplementary Table 1.** Search keywords and search line

|  | **Query and search Keywords** |
| --- | --- |
| ***Intervention*** | “coffee” OR “caffeine” OR “caffeinated” OR “decaffeinated” |
| ***Outcomes*** | “Breast cancer” OR “Breast malignancy” OR “Breast carcinoma” OR “Breast adenocarcinoma” OR “Breast tumor” |
| **Search strategy** | (“coffee” OR “caffeine” OR “caffeinated” OR “decaffeinated”) **AND** (“Breast cancer” OR “Breast malignancy” OR “Breast carcinoma” OR “Breast adenocarcinoma” OR “Breast tumor”) |

**Supplementary Table 2.** Search line and query box in databases

| **Databases** | **Search line** | **#** |
| --- | --- | --- |
| ***PubMed*** | ("coffee"[Title/Abstract] OR "caffeine"[Title/Abstract] OR "caffeinated"[Title/Abstract] OR "decaffeinated"[Title/Abstract]) AND ("Breast cancer"[Title/Abstract] OR "Breast malignancy"[Title/Abstract] OR "Breast carcinoma"[Title/Abstract] OR "Breast adenocarcinoma"[Title/Abstract] OR "Breast tumor"[Title/Abstract]) | 327 |
| ***WoS*** | Results for “coffee” OR “caffeine” OR “caffeinated” OR “decaffeinated” (Topic) AND “Breast cancer” OR “Breast malignancy” OR “Breast carcinoma” OR “Breast adenocarcinoma” OR “Breast tumor” (Topic) | 670 |
| ***Scopus*** | (TITLE-ABS-KEY ("coffee" OR “caffeine” OR “caffeinated" OR “decaffeinated”) AND TITLE-ABS-KEY (“Breast cancer” OR “Breast malignancy” OR “Breast carcinoma” OR “Breast adenocarcinoma” OR “Breast tumor”)) | 875 |
| ***All*** | PubMed (n = 327), ISI Web of Science (n = 670), Scopus (n = 875) | 1872 |
